# Advancing dengue vaccine development: Challenges, innovations, and the path toward global protection

**DOI:** 10.1002/ped4.70005

**Published:** 2025-04-15

**Authors:** Ran Wang, Bridget Kim, Hridesh Mishra, Kevin C. Kain

**Affiliations:** ^1^ Laboratory of Infection and Virology Beijing Pediatric Research Institute Beijing Children's Hospital Capital Medical University National Center for Children's Health Beijing China; ^2^ Research Unit of Critical Infection in Children, 2019RU016 Chinese Academy of Medical Sciences Beijing China; ^3^ Sandra A. Rotman (SAR) Laboratories Sandra Rotman Centre for Global Health University Health Network‐Toronto General Hospital Toronto Canada; ^4^ Department of Medicine Tropical Disease Unit Division of Infectious Diseases University of Toronto Toronto Canada; ^5^ Department of Experimental Therapeutics University Health Network‐Toronto General Hospital Toronto Canada; ^6^ Faculty of Medicine University of Toronto Toronto Canada

**Keywords:** Antibody‐dependent enhancement, Dengue fever, Dengue vaccine, Tetravalent protection, Vaccine platforms

## Abstract

Dengue fever remains a significant global health threat, placing nearly half of the world's population at risk. Despite decades of research, developing an effective dengue vaccine continues to face multiple challenges, including antibody‐dependent enhancement (ADE), serotype‐specific efficacy, and logistical barriers to vaccine delivery. This review provides a comprehensive analysis of the historical and current landscape of dengue vaccine development, focusing on CYD‐TDV, TAK‐003, and Butantan‐DV. It examines their efficacy, safety profiles, and limitations, particularly in achieving balanced quadrivalent protection. Additionally, this review highlights key areas for future research, including the impact of ADE, advancements in vaccine platforms, the need for region‐specific vaccine formulations, and the integration of vaccination into broader dengue prevention strategies. Ultimately, sustained investment and global collaboration are crucial for achieving the goal of a dengue‐free world.

## Graphical abstract

Dengue vaccine development faces challenges, including antibody‐dependent enhancement and serotype‐specific immunity. This review highlights licensed vaccines, CYD‐TDV, TAK‐003, and Butantan‐DV, and explores strategies for achieving effective multivalent protection.

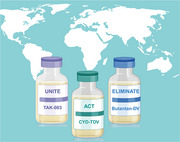

## INTRODUCTION

Dengue fever is a major global health threat, putting almost half of the world's population at risk. Efforts to develop an effective dengue vaccine have faced many challenges since the early 20th century. Initial rudimentary attempts, such as the use of ox bile to attenuate the dengue virus (DENV) and the chemical processing of infected mosquitoes, have evolved into sophisticated scientific endeavors. Despite these advancements, the quest for a viable dengue vaccine is ongoing, driven by the urgent need to protect millions of individuals in endemic regions. This Viewpoint explores the status and implications of current dengue vaccines, including CYD‐TDV, TAK‐003, and Butantan‐DV, within the broader context of dengue vaccine development and the challenges posed by the disease.

## DENGUE IMMUNOLOGY AND EPIDEMIOLOGY

DENV is a flavivirus with four serotypes (DENV‐1 to DENV‐4) that elicit both protective and pathogenic immune responses. Primary infection typically induces a serotype‐specific immune response, while secondary heterotypic infections can lead to enhanced disease severity due to antibody‐dependent enhancement (ADE). The immune response involves both humoral (antibody‐mediated) and cellular (T‐cell mediated) mechanisms, with cross‐reactive memory B and T cells playing a role in both protection and pathogenesis. Epidemiologically, dengue is endemic in over 100 countries, with periodic outbreaks influenced by serotype dynamics, climate, and population immunity. Understanding these factors is crucial for vaccine development and implementation.

## MECHANISMS OF SEVERE DENGUE IMMUNOPATHOLOGY

Severe dengue disease is primarily driven by ADE, which occurs in heterotypic secondary infections and in infants with maternally derived non‐neutralizing antibodies. ADE is initiated when immune complexes of DENV and IgG antibodies bind to Fcγ receptors (FcγR) on myeloid cells, suppressing antiviral defenses and enhancing viral replication.^1^ This FcγR‐mediated immune suppression has been validated in experimental models.^2^ Additionally, elevated levels of dengue NS1 protein, a key virulence factor, have been associated with severe disease.^3^ Elevated NS1 concentrations contribute to endothelial dysfunction and increased vascular permeability, which are central to severe dengue pathology.

## HISTORICAL ATTEMPTS AT DENGUE VACCINE DEVELOPMENT

Early dengue vaccine development efforts included the use of ox bile to attenuate the DENV, along with other rudimentary methods such as chemical processing of DENV‐infected mosquitoes in the late 1920s.^4^ However, the quest for efficacious dengue vaccines remains a challenge that continues to confound scientific inquiry. The urgent market demand and vast at‐risk population have intensified efforts to develop an effective dengue vaccine, despite ongoing challenges. Here we discuss the status of CYD‐TDV, TAK‐003, and Butantan‐DV vaccines.

## INSIGHTS FROM CYD‐TDV: THE FIRST APPROVED VACCINE

Sanofi Pasteur's CYD‐TDV (Dengvaxia) was the first licensed dengue vaccine, demonstrating efficacy in phase III trials. However, it was later found that while CYD‐TDV induces neutralizing antibodies against all four DENV serotypes, it predominantly elicits homotypic neutralizing antibodies against DENV‐4, with reduced cross‐protection against DENV‐1, DENV‐2, and DENV‐3.^5^, ^6^ The design of CYD‐TDV, which utilizes a yellow fever 17D (YF17D) backbone, may have resulted in over‐attenuation of the DENV‐1, DENV‐2, and DENV‐3 components, leading to suboptimal immune responses and lower cross‐neutralization.^6^ Studies evaluating viremia in vaccinated individuals have corroborated this hypothesis, indicating limited replication of certain serotypes and an imbalanced immune response. The withdrawal of CYD‐TDV from widespread use was driven primarily by two key factors: (1) The requirement for serological testing prior to vaccination: Due to the increased risk of severe dengue and hospitalization in seronegative vaccine recipients, CYD‐TDV was recommended only for individuals with confirmed prior DENV infection.^5^ This seroprevalence screening requirement significantly limited its practical implementation in many endemic settings. (2) The requirement for a three‐dose schedule over 12 months: Unlike single‐dose or two‐dose vaccines, CYD‐TDV's three‐dose regimen posed logistical challenges, particularly in resource‐limited settings where long‐term vaccine adherence is difficult to achieve. The extended dosing schedule also impacted vaccine uptake and compliance, further restricting its effectiveness at the population level. These limitations, compounded by public controversy and adverse events during its initial rollout, have led to the vaccine being largely withdrawn from routine use. Despite its setbacks, CYD‐TDV's development has provided important insights into dengue immunity and vaccine design, emphasizing the need for balanced quadrivalent protection and improved immunogenicity across all four DENV serotypes. Consequently, the vaccine has struggled to achieve balanced quadrivalent protection, particularly in seronegative populations. Despite this, CYD‐TDV is recommended by the World Health Organization (WHO) for individuals aged 9–45 years with prior DENV infection,^7^ with narrower recommendations in the United States, where it is advised only for individuals aged 9–16 years with confirmed dengue infection in endemic areas.^8^ The abrupt revocation of CYD‐TDV's approval in the Philippines further underscored the challenges of developing dengue vaccines that are both safe and broadly applicable.^9^

## TAK‐003: PROGRESS AND CHALLENGES

Takeda's TAK‐003 dengue vaccine represents an alternative approach, evaluated over a 4.5‐year phase III trial in eight dengue‐endemic nations in Asia and Latin America. The vaccine demonstrated an overall efficacy of 61.2% against virologically confirmed dengue (VCD) and 84.1% against hospitalized VCD.^10^ Its protection was particularly robust for DENV‐1 and DENV‐2 in seropositive individuals, but efficacy in seronegative populations was limited to DENV‐1 and DENV‐2, with no significant protection observed for DENV‐3 or DENV‐4 due to an insufficient number of cases for evaluation. TAK‐003's two‐dose regimen provides sustained protection, but logistical challenges remain for its implementation in resource‐limited settings.

## BUTANTAN‐DV: SIMPLIFYING VACCINE DELIVERY

Recent findings suggest that Butantan‐DV may offer advantages in vaccine delivery due to its single‐dose regimen, which simplifies implementation in regions with limited healthcare infrastructure. A 2‐year analysis reported an overall efficacy of 73.6% in sero‐naïve individuals and 89.2% in those with prior dengue exposure, with protection against DENV‐1 (89.5%) and DENV‐2 (69.6%).^11^ A more relevant comparison between TAK‐003 and Butantan‐DV should focus on sero‐naïve individuals and younger children, as the safety concerns for CYD‐TDV were primarily identified in sero‐naïve children aged 2–5 years. The efficacy of TAK‐003 in sero‐naïve children aged 4–5 years was 55.9%,^12^ whereas the efficacy of Butantan‐DV in sero‐naïve children aged 2–6 years was 73.4%.^11^ These findings suggest that Butantan‐DV may provide stronger early protection in the most vulnerable age group. Long‐term data further support Butantan‐DV's protective efficacy beyond the initial 2‐year period. In a >3‐year follow‐up study, Butantan‐DV demonstrated an 89% reduction in severe dengue and dengue with warning signs.^13^ Similarly, extended follow‐up data for TAK‐003 showed variability in efficacy across serotypes and age groups, reinforcing the need for continued long‐term evaluation.^14^ Although the Butantan‐DV vaccine demonstrated promising results, its efficacy against DENV‐3 and DENV‐4 could not be established in the Phase III clinical trial in Brazil due to the absence of these serotypes during the study period.^11^ Further post‐licensure surveillance will be necessary to determine their long‐term effectiveness in real‐world settings. In addition to efficacy, administration schedules also play a crucial role in vaccine deployment. Butantan‐DV's single‐dose regimen presents a potential logistical advantage over TAK‐003, which requires two doses administered three months apart. In resource‐limited settings, adherence to multi‐dose regimens may be challenging, making single‐dose vaccines a more practical option. However, additional research is needed to confirm whether Butantan‐DV's single‐dose formulation provides sustained long‐term immunity comparable to TAK‐003's booster approach.

Importantly, current dengue vaccines have demonstrated efficacy in reducing severe and fatal dengue in clinical trials. However, their impact on individuals older than 60 years remains unknown, as they have not been evaluated in this age group. While the removal of age restrictions for some vaccine administrations may broaden access, further research is needed to determine whether these vaccines can effectively lower mortality rates in individuals aged over 60. In this demographic, managing dengue‐related plasma leakage and dehydration is particularly challenging due to age‐related changes in vascular integrity and comorbidities that complicate fluid therapy adjustments.

## CHALLENGES OF ADE IN DENGUE VACCINES

Since the inception of dengue vaccine development, an inescapable discourse has revolved around the potential adverse effects of severe dengue following vaccination. ADE may be the main cause of severe dengue after vaccination, a concern often cited by vaccine critics. Any discussion of ADE must consider the structural design of these vaccines, particularly their chimeric backbones. CYD‐TDV utilizes the YF17D backbone to express dengue envelope proteins, while TAK‐003 uses a DENV‐2 backbone to express envelope genes from other DENV serotypes. The ability of each vaccine component to infect and replicate within the host plays a crucial role in determining serotype‐specific immune responses. While vaccine design can contribute to this by influencing the degree of attenuation, the primary determinant is the replication competency of the live‐attenuated virus.

Central to this theory is the assumption that antibodies constitute the sole precipitant of this outcome, necessitating sub‐neutralizing concentrations of DENV antibodies. Based on experiences with CYD‐TDV, individuals with no serum DENV antibodies (i.e., individuals with no history of prior dengue infection) were more likely to develop symptoms, including severe dengue, after vaccination. This is likely due to the vaccine's inability to induce sufficient protective immunity in the absence of a pre‐existing immune response generated by prior dengue exposure. Conversely, secondary dengue infections typically elicit a broader immune response, including highly neutralizing antibodies and CD8^+^ T cell responses targeting conserved epitopes across all four serotypes.^15^, ^16^ Studies have demonstrated that secondary infections induce a significant expansion of cross‐reactive, highly neutralizing antibodies and robust T‐cell responses, which contribute to enhanced viral clearance and reduced risk of severe disease.^17^, ^18^ This expanded immune response after a second infection may contribute to achieving neutralizing titers sufficient to reduce the risk of severe disease.

ADE is primarily driven by cross‐reactive, non‐neutralizing antibodies that recognize highly conserved epitopes on the DENV envelope (E) protein.^19^ The seminal work by Kliks et al.^20^ first identified maternal antibody titers as a key risk factor for severe dengue, providing early evidence for ADE in infants. Notably, the fusion loop epitope (FLE) in domain II of the E protein is a key target of such antibodies.^21^ While highly conserved across all four DENV serotypes, antibodies against the FLE often fail to neutralize heterotypic infections effectively, instead facilitating FcγR‐mediated viral entry into myeloid cells.^22^ FcγRs, particularly FcγRIIa and FcγRIII, play a central role in ADE by enabling the internalization of antibody‐opsonized virions into monocytes, macrophages, and dendritic cells.^23^ This process bypasses classical viral entry pathways, allowing for increased viral replication and systemic dissemination. Furthermore, FcγR engagement can trigger downstream signaling cascades that suppress type I interferon responses, weakening antiviral immunity and contributing to increased disease severity.^24^

Understanding the role of conserved epitopes and FcγR signaling in ADE is crucial for dengue vaccine development. Vaccine candidates must carefully balance the induction of neutralizing antibodies against all four DENV serotypes while minimizing the risk of sub‐neutralizing, cross‐reactive antibodies that may enhance ADE. Future vaccine strategies should consider antigen design approaches that limit the immunogenicity of the FLE while promoting the generation of protective, serotype‐specific responses. Conversely, individuals with prior exposure to DENV will experience the induction of higher and more enduring antibody levels post‐vaccination, acting as a booster. As an example, in dengue‐endemic regions, the incidence of dengue among infants less than 1 year is markedly elevated. This phenomenon is attributed, in part, to the attenuation of maternal DENV antibodies to sub‐neutralizing levels, which may enhance susceptibility to infection.^25^ Given the placenta's complex structure and function that effectively filters and blocks most cells, pathogens, and large molecules while selectively transporting nutrients, antibodies, and some small molecules, it highlights the direct influence of antibodies. As maternal antibodies wane, newborns may face increased susceptibility to severe dengue fever or hospitalized dengue fever.

In addition to the effects of ADE, the possibility of serotype replacement circulation in endemic regions cannot be ruled out. The return of a DENV serotype that has not circulated for a long time may render a considerable number of children relatively susceptible, which could also be one of the factors contributing to increased susceptibility. The “original antigenic sin (OAS)” theory (also known as “immune imprinting”) suggests that an individual's initial infection largely determines their protection against future related serotypes, while the impact on ADE cannot be overlooked. Since ADE is primarily driven by pre‐existing antibodies, OAS in antibody responses to DENV may lead to the production of low‐affinity antibodies, increasing the risk of ADE during secondary infections.^26^ A recent study reports that the severity of dengue fever is associated with the specific sequence of infecting serotypes and the antigenic distance between primary and secondary infections, with the risk being highest when the antigenic distance is at a moderate level.^27^ In fact, severe dengue is more frequently observed during secondary infections with heterologous serotype DENV but tends to decline in prevalence during tertiary or quaternary infections. This pattern suggests that multiple infections expand the immune repertoire, promoting the development of broadly neutralizing antibodies and enhanced immune regulation, which collectively reduce the risk of severe disease in later infections.^28^

Furthermore, elucidation of the non‐neutralizing (non‐protective) threshold remains an urgent priority, yet consensus remains elusive. However, a truly efficacious dengue vaccine should elicit homotypic neutralizing antibodies to all four serotypes, thereby mitigating the risk of severe disease upon subsequent infections.^29^ Epidemiological studies indicate that reinfection with the same serotype rarely leads to symptomatic illness, as the initial infection induces robust homotypic neutralizing antibodies that prevent reinfection.^17^ The correlation between homotypic neutralizing antibody levels and vaccine efficacy, as observed for CYD‐TDV, TAK‐003, and TV003, underscores the importance of inducing a balanced immune response across all serotypes.^18^ This issue should be addressed concurrently with the ongoing phase IV efficacy studies. Additionally, why is ADE more commonly observed between different DENV serotypes or in secondary infections with heterologous flaviviruses, whereas it is relatively rare in other viruses?^30^, ^31^ One explanation pertains to inherent differences in viral profiles, underscored by specialized ADE “codes” intrinsic to DENV, such as the FLE on envelope domain II.^21^ It may also be due to the inability of the aforementioned virus to replicate in cells bearing Fcγ receptors, in addition to factors related to antigenic variability.

Overall, ADE issues in the real world may only be revealed through efficacy studies of phase IV clinical trials of vaccines. This includes post‐marketing periods and all inherent events, especially those related to safety and drug vigilance, which often manifest several years after the campaign. Current clinical data can only conservatively suggest differences in protective rates across all serotypes for CYD‐TDV, TAK‐003, and Butantan‐DV.

## PURSUING MULTIVALENT DENGUE VACCINES: PATHWAYS AND CONSIDERATIONS

All stakeholders share the aspiration for a multivalent dengue vaccine capable of effectively protecting against all four DENV serotypes. The attainment of quadrivalent equilibrium protection remains an ambitious goal, challenging to achieve despite the demonstrated potential of each vaccine candidate during clinical trials. CYD‐TDV, as the vanguard dengue vaccine, provides a blueprint for comparative analyses and imparts important lessons for subsequent vaccine design enhancements. In light of the discussions surrounding ADE, the imperative for quadrivalent vaccines demands careful consideration, as the vaccine must induce serotype‐specific neutralizing antibodies against all four DENV serotypes to mitigate the risk of ADE. Epidemiological studies suggest that symptomatic reinfection with the same serotype is rare due to robust immunity from primary infections, which generates homologous neutralizing antibodies and memory responses for effective viral clearance.^32^ Given these immunological principles, dengue vaccines do not need to be tailored to specific regional serotype distributions, as achieving serotype‐specific formulations for different locations would be impractical for vaccine manufacturers. Instead, the primary goal of dengue vaccination is to induce a durable immune response that mitigates disease severity across all serotypes, rather than completely preventing infection. While serotype shifts and periodic outbreaks may occur, effective multivalent vaccines should provide broad protection by ensuring robust neutralizing antibody responses against all four DENV serotypes. Although the WHO's annual coordination of the global influenza surveillance network provides a framework for addressing globally circulating pathogens, this approach cannot be directly applied to dengue due to fundamental differences between the two diseases. Unlike influenza, where antigenic drift can lead to globally dominant strains, dengue serotype distribution is far more unpredictable, influenced by factors such as past outbreaks, transmission dynamics, and the time interval since the last epidemic. These differences underscore the importance of understanding regional serotype circulation trends for surveillance and vaccine deployment strategies, rather than modifying the vaccine formulation itself. The widespread cocirculation of all four dengue serotypes further reinforces the necessity of a tetravalent vaccine that provides durable protection across serotypes. However, challenges remain in optimizing vaccine dosage and immune response balance to achieve consistent efficacy. In this context, single‐dose vaccines remain an attractive option, particularly in an era of increasing vaccine hesitancy, as they improve accessibility and compliance in endemic settings.

## LOOKING AHEAD: INNOVATIVE PATHWAYS TO ERADICATION

The banner of World Neglected Tropical Diseases Day 2024 carries the imperative message “Unite, Act, Eliminate” echoing the call for concerted efforts in the development of dengue vaccines, a critical aspect of addressing this neglected disease. Unite: The international community must coalesce, fostering collaboration among governments, academia, the medical sector, non‐governmental organizations, and vaccine manufacturers. Enhancing the sharing of molecular epidemiological data and resources, along with vaccine design experiences, while mitigating redundant endeavors, is paramount. Through unified action, the efficiency and progress of dengue vaccine research and development can be advanced. Act: Embracing a diverse array of vaccine development platforms, notably messenger RNA (mRNA) vaccines, necessitates an increased investment of financial, human, and technical resources to expedite the pace of dengue vaccine research and development. However, while mRNA technology demonstrated remarkable success during the coronavirus disease 2019 pandemic, its application to dengue poses unique challenges that warrant careful evaluation. A major challenge is the need to elicit balanced, high‐titer neutralizing antibodies against all four DENV serotypes simultaneously. Unlike severe acute respiratory syndrome coronavirus 2, DENV consists of four antigenically distinct serotypes, requiring a quadrivalent immune response to achieve comprehensive protection. To avoid ADE, several key factors must be optimized in mRNA vaccine design: (1) Antigen selection and codon optimization: the mRNA sequences encoding the E protein must be designed to preferentially induce high‐affinity, serotype‐specific neutralizing antibodies while minimizing cross‐reactive, non‐neutralizing antibodies that could facilitate ADE. (2) Lipid nanoparticle delivery and immunogenicity enhancement: the formulation must ensure efficient antigen expression and optimal immune activation, as some mRNA vaccines may require adjuvants or modified delivery mechanisms to enhance immunogenicity against flaviviruses.^33^ (3) T‐cell response modulation: mRNA vaccines should be designed to activate broad CD8^+^ and CD4^+^ T‐cell responses, ensuring not only humoral but also cellular immunity against all four DENV serotypes. (4) Durability of protection and booster strategies: unlike live‐attenuated vaccines that may mimic natural infection, mRNA vaccines might require booster doses to sustain protective immunity, particularly in populations with prior DENV exposure. Further, the WHO has outlined critical considerations for developing mRNA‐based candidates in resource‐limited settings, emphasizing the importance of infrastructure, scalability, and cost‐effectiveness in ensuring equitable access.^34^ Proactive engagement and resource allocation are indispensable in this pursuit. Eliminate: Integrating dengue vaccine research and development into comprehensive dengue fever control and prevention plans is imperative. Additionally, concerted efforts should be directed toward bolstering research on DENV transmission pathways, enhancing prevention and control measures, and mitigating mosquito‐borne transmission. These include initiatives such as the elimination of breeding sites and improvements in environmental sanitation. Ultimately, the goal is to eradicate the spread of dengue fever globally. To achieve these goals, several future research directions are critical for advancing dengue vaccine efficacy and safety: (1) Phase IV studies of vaccine‐induced ADE are important: Conducting Phase IV studies is essential to evaluate ADE, focusing on FcγR‐mediated suppression and NS1‐induced endothelial damage to refine vaccine strategies. Additionally, expanding vaccine research to include alternative immunization strategies and further epidemiological studies will enhance our understanding of optimal dengue prevention methods. (2) Further development of diverse vaccine platforms for dengue vaccines is valuable: Expanding the scope of dengue vaccine development to include varied platforms offers the potential for improved efficacy and adaptability across different populations and DENV serotypes, addressing current vaccine limitations. (3) Tailoring vaccine composition to population needs and integrating strain information: Customizing dengue vaccine formulations to reflect regional DENV strain prevalence could optimize efficacy. Additionally, integrating vaccination efforts into comprehensive dengue prevention programs will enhance disease control measures. Looking ahead, sustained investment in research, innovative vaccine modalities, and comprehensive disease management strategies will be pivotal in realizing the vision of a dengue‐free world.

## CONFLICT OF INTEREST

The authors declare no conflict of interest.

## References

[1] Halstead SB , Mahalingam S , Marovich MA , Ubol S , Mosser DM . Intrinsic antibody‐dependent enhancement of microbial infection in macrophages: disease regulation by immune complexes. Lancet Infect Dis. 2010;10:712‐722. DOI: 10.1016/S1473-3099(10)70166-3 20883967 PMC3057165

[2] Williams KL , Zompi S , Beatty PR , Harris E . A mouse model for studying dengue virus pathogenesis and immune response. Ann N Y Acad Sci. 2009;1171:E12‐23. DOI: 10.1111/j.1749-6632.2009.05057.x 19751398

[3] Puerta‐Guardo H , Glasner DR , Harris E . Dengue virus NS1 disrupts the endothelial glycocalyx, leading to hyperpermeability. PLoS Pathog. 2016;12:e1005738. DOI: 10.1371/journal.ppat.1005738 27416066 PMC4944995

[4] Simons JS , St John JH , Reynolds FHK . Experimental studies of dengue. Philipp J Sci. 1931;44:1‐252.

[5] Sridhar S , Luedtke A , Langevin E , Zhu M , Bonaparte M , Machabert T , et al. Effect of dengue serostatus on dengue vaccine safety and efficacy. N Engl J Med. 2018;379:327‐340. DOI: 10.1056/NEJMoa1800820 29897841

[6] Henein S , Swanstrom J , Byers AM , Moser JM , Shaik SF , Bonaparte M , et al. Dissecting antibodies induced by a chimeric yellow fever‐dengue, live‐attenuated, tetravalent dengue vaccine (CYD‐TDV) in naive and dengue‐exposed individuals. J Infect Dis. 2017;215:351‐358. DOI: 10.1093/infdis/jiw576 27932620 PMC6392503

[7] Dengue vaccine: WHO position paper, September 2018 ‐ Recommendations. Vaccine. 2019;37:4848‐4849. DOI: 10.1016/j.vaccine.2018.09.063 30424888

[8] Paz‐Bailey G , Adams L , Wong JM , Poehling KA , Chen WH , McNally V , et al. Dengue vaccine: recommendations of the Advisory Committee on Immunization Practices, United States, 2021. MMWR Recomm Rep. 2021;70:1‐16. DOI: 10.15585/mmwr.rr7006a1 PMC8694708 34978547

[9] Thomas SJ . Is new dengue vaccine efficacy data a relief or cause for concern? NPJ Vaccines. 2023;8:55. DOI: 10.1038/s41541-023-00658-2 37061527 PMC10105158

[10] Tricou V , Yu D , Reynales H , Biswal S , Saez‐Llorens X , Sirivichayakul C , et al. Long‐term efficacy and safety of a tetravalent dengue vaccine (TAK‐003): 4.5‐year results from a phase 3, randomised, double‐blind, placebo‐controlled trial. Lancet Glob Health. 2024;12:e257‐e270. DOI: 10.1016/S2214-109X(23)00522-3 38245116

[11] Kallás EG , Cintra M , Moreira JA , Patiño EG , Braga PE , Tenório J , et al. Live, attenuated, tetravalent Butantan‐dengue vaccine in children and adults. N Engl J Med. 2024;390:397‐408. DOI: 10.1056/NEJMoa2301790 38294972

[12] López‐Medina E , Biswal S , Saez‐Llorens X , Borja‐Tabora C , Bravo L , Sirivichayakul C , et al. Efficacy of a dengue vaccine candidate (TAK‐003) in healthy children and adolescents 2 years after vaccination. J Infect Dis. 2022;225:1521‐1532. DOI: 10.1093/infdis/jiaa761 33319249 PMC9071282

[13] Nogueira ML , Cintra M , Moreira JA , Patiño EG , Braga PE , Tenório J , et al. Efficacy and safety of Butantan‐DV in participants aged 2‐59 years through an extended follow‐up: results from a double‐blind, randomised, placebo‐controlled, phase 3, multicentre trial in Brazil. Lancet Infect Dis. 2024;24:1234‐1244. DOI: 10.1016/S1473-3099(24)00376-1 39116904

[14] Rivera L , Biswal S , Sáez‐Llorens X , Reynales H , López‐Medina E , Borja‐Tabora C , et al. Three‐year efficacy and safety of Takeda's dengue vaccine candidate (TAK‐003). Clin Infect Dis. 2022;75:107‐117. DOI: 10.1093/cid/ciab864 34606595 PMC9402653

[15] Coloma J , Harris E . Broad and strong: the ultimate antibody to dengue virus. Nat Immunol. 2015;16:135‐137. DOI: 10.1038/ni.3081 25594456

[16] Weiskopf D , Cerpas C , Angelo MA , Bangs DJ , Sidney J , Paul S , et al. Human CD8+ T‐cell responses against the 4 dengue virus serotypes are associated with distinct patterns of protein targets. J Infect Dis. 2015;212:1743‐1751. DOI: 10.1093/infdis/jiv289 25980035 PMC4633759

[17] Dejnirattisai W , Wongwiwat W , Supasa S , Zhang X , Dai X , Rouvinski A , et al. A new class of highly potent, broadly neutralizing antibodies isolated from viremic patients infected with dengue virus. Nat Immunol. 2015;16:170‐177. DOI: 10.1038/ni.3058 25501631 PMC4445969

[18] Weiskopf D , Angelo MA , de Azeredo EL , Sidney J , Greenbaum JA , Fernando AN , et al. Comprehensive analysis of dengue virus‐specific responses supports an HLA‐linked protective role for CD8+ T cells. Proc Natl Acad Sci U S A. 2013;110:E2046‐2053. DOI: 10.1073/pnas.1305227110 23580623 PMC3670335

[19] Sarker A , Dhama N , Gupta RD . Dengue virus neutralizing antibody: a review of targets, cross‐reactivity, and antibody‐dependent enhancement. Front Immunol. 2023;14:1200195. DOI: 10.3389/fimmu.2023.1200195 37334355 PMC10272415

[20] Kliks SC , Nimmanitya S , Nisalak A , Burke DS . Evidence that maternal dengue antibodies are important in the development of dengue hemorrhagic fever in infants. Am J Trop Med Hyg. 1988;38:411‐419. DOI: 10.4269/ajtmh.1988.38.411 3354774

[21] Kotaki T , Kurosu T , Grinyo‐Escuer A , Davidson E , Churrotin S , Okabayashi T , et al. An affinity‐matured human monoclonal antibody targeting fusion loop epitope of dengue virus with in vivo therapeutic potency. Sci Rep. 2021;11:12987. DOI: 10.1038/s41598-021-92403-9 34155267 PMC8217507

[22] Castanha PMS , Erdos G , Watkins SC , Falo LD Jr , Marques ETA , Barratt‐Boyes SM . Reciprocal immune enhancement of dengue and Zika virus infection in human skin. JCI Insight. 2020;5:e133653. DOI: 10.1172/jci.insight.133653 31910161 PMC7098780

[23] Bournazos S , Gupta A , Ravetch JV . The role of IgG Fc receptors in antibody‐dependent enhancement. Nat Rev Immunol. 2020;20:633‐643. DOI: 10.1038/s41577-020-00410-0 32782358 PMC7418887

[24] Bournazos S , Wang TT , Dahan R , Maamary J , Ravetch JV . Signaling by antibodies: recent progress. Annu Rev Immunol. 2017;35:285‐311. DOI: 10.1146/annurev-immunol-051116-052433 28446061 PMC5613280

[25] O'Driscoll M , Buddhari D , Huang AT , Waickman A , Kaewhirun S , Iamsirithaworn S , et al. Maternally derived antibody titer dynamics and risk of hospitalized infant dengue disease. Proc Natl Acad Sci U S A. 2023;120:e2308221120. DOI: 10.1073/pnas.2308221120 37774093 PMC10576102

[26] Midgley CM , Bajwa‐Joseph M , Vasanawathana S , Limpitikul W , Wills B , Flanagan A , et al. An in‐depth analysis of original antigenic sin in dengue virus infection. J Virol. 2011;85:410‐421. DOI: 10.1128/JVI.01826-10 20980526 PMC3014204

[27] Wang L , Huang AT , Katzelnick LC , Lefrancq N , Escoto AC , Duret L , et al. Antigenic distance between primary and secondary dengue infections correlates with disease risk. Sci Transl Med. 2024;16:eadk3259. DOI: 10.1126/scitranslmed.adk3259 38657027 PMC12336440

[28] Halstead SB . Three dengue vaccines ‐ what now? N Engl J Med. 2024;390:464‐465. DOI: 10.1056/NEJMe2314240 38294979

[29] de Silva AM , Harris E . Which dengue vaccine approach is the most promising, and should we be concerned about enhanced disease after vaccination? The path to a dengue vaccine: learning from human natural dengue infection studies and vaccine trials. Cold Spring Harb Perspect Biol. 2018;10:a029371. DOI: 10.1101/cshperspect.a029371 28716891 PMC5983190

[30] Estofolete CF , Versiani AF , Dourado FS , Milhim B , Pacca CC , Silva G , et al. Influence of previous Zika virus infection on acute dengue episode. PLoS Negl Trop Dis. 2023;17:e0011710. DOI: 10.1371/journal.pntd.0011710 37943879 PMC10662752

[31] Coish JM , MacNeil LA , MacNeil AJ . The SARS‐CoV‐2 antibody‐dependent enhancement façade. Microbes Infect. 2024:105464. DOI: 10.1016/j.micinf.2024.105464 39662700

[32] Khanam A , Gutiérrez‐Barbosa H , Lyke KE , Chua JV . Immune‐mediated pathogenesis in dengue virus infection. Viruses. 2022;14:2575. DOI: 10.3390/v14112575 36423184 PMC9699586

[33] Chaudhary N , Weissman D , Whitehead KA . mRNA vaccines for infectious diseases: principles, delivery and clinical translation. Nat Rev Drug Discov. 2021;20:817‐838. DOI: 10.1038/s41573-021-00283-5 34433919 PMC8386155

[34] Gsell PS , Giersing B , Gottlieb S , Wilder‐Smith A , Wu L , Friede M . Key considerations for the development of novel mRNA candidate vaccines in LMICs: a WHO/MPP mRNA Technology Transfer Programme meeting report. Vaccine. 2023;41:7307‐7312. DOI: 10.1016/j.vaccine.2023.10.027 37949751

